# Medical Profession

**Published:** 1875-02

**Authors:** 


### HALL'S

### MEDICAL ADVISER.

(ALL ARTICLES WRITTEN BY THE EDITOR.)

FEBRUARY, 1875.

### THE MEDICAL PROFESSION.

WITHIN the last two or three hundred years, the observations of physicians into the causes of disease, and their dissemination of information as to the methods of removing these causes, and the laws of health and life, have resulted in a very great part in doubling the duration of human existence. In the days of Queen Elizabeth, when Bacon, and Shakespeare, and Ben Johnson, and other great names made the age famous, the average duration of life in the more civilized nations was about twenty-one years. Now it is over forty.

Within the last third of a century, greater progress has been made into the causes of disease as affecting communities, than in the preceding centuries; and within the last dozen or two years, more has been written as a result of definite and specific investigation of the causes of sickness to families and communities, than perhaps in all previous medical history.

These researches have been made in connection with drainage, sewerage, sites for houses, modes of building and warming, and water supply; and these have become so important, so essential to the comfort and health of individuals, families, and whole districts, that the attention of public men, high in office and influence, has been called to it; and not only so, some of them have become so impressed with its importance, that they have thought it well, when opportunity offered, to commend these studies to the public. Governor Dix, in his late address to the Albany Medical College, said:

"It may seem like presumption on my part to make a suggestion for your consideration, with no more knowledge of the medical profession than I need to write my own prescriptions in apothecary's Latin; and yet I have some claim to be heard, as I had in my early life many years of disordered health, which I overcame effectually by a careful investigation of the cause, and by a persevering adherence to the laws which nature prescribes for our government, and which I was compelled to learn by experience for want of proper professional counsel. The suggestion I desire to make is this: that while chemistry and physiology have opened to the department of therapeutics a field of investigation unknown to the ancients, the principles of hygiene have not been studied with the same care, except during the last few years on a large scale—in their application to communities and aggregations of men, rather than to single individuals. The theory of this system is to prevent disease instead of curing it, and the former is as much the province of the physician as the latter. This was one of the chief aims of that celebrated—I might say that unrivaled—school of Salernum, which held undisputed dominion over Europe throughout the middle ages in all that related to medical science and practice."

And Ex-Governor Hoffman subsequently, in referring to the virtues that pertain in a special degree to the medical profession, said: "The main source of your livelihood, of your pecuniary gains, is sickness among the people. The more disease, the more calls for you—yet everywhere medical men are devoting their best thoughts and energies to preventing disease, and to spreading abroad among the people a knowledge of the laws of health, so as to enable them more and more to do without your professional services. In the gratuitous investigation by physicians of the causes of epidemic and other diseases, with a view of their prevention, some of the noblest of lives are sacrificed. Such men are real heroes, although not so conspicuous in popular history as those who fall in battle between contending armies. They die for their fellow-men in a struggle in which science and death contend against each other for the mastery, and the world hardly knows that the contest is going on. All honor to them and to the profession to which they belong."

Except by the well-informed, the nobility of the medical profession has never been fully appreciated. Professional honor calls the physician to the bedside of the poor as well as the rich, day or night, Summer or Winter. No class of men gives half as much personal service or make half the pecuniary sacrifices as medical men. In one respect, their nobility of character stands out in striking contrast with that of the great mass of men. When they find a new and valuable remedy for any human malady, it is at once made common property; it is communicated to medical journals, that it may be known throughout the world, and used for the benefit of sick and suffering humanity. In all other departments of human life, a valuable secret is locked up in the breast of the discoverer, and turned into money, or its monopoly is secured by a patent for the use of the individual. If the uses of ether, or nitrous oxyde, or chloroform, in surgery, had been concealed, their discoverers would, within a few years, have been the richest men on the globe. They told all they knew, and died poor.

Physicians are very careful in announcing new remedies. They are not easily convinced of the efficacy of new things, new drugs, new combinations. They prefer to make a thorough trial under the varying conditions of age, and sex, and season, and condition of life. Very often five, ten, or fifteen years pass before the success has been sufficiently uniform to warrant them in their own estimation in making their supposed discoveries public. Their professional pride makes them afraid of being premature.

If an ordinary person uses a remedy, and immediate, complete and permanent cure follows, he is in ecstacies, and for the next man he meets with having an ailment at all alike his own, he exhausts his language of its adjectives in praise of the remedy he used, forgetting that he might have got well if he had done nothing, for he might have taken the medicine at the crisis of the disease, when it was "upon the turn," had exhausted itself, and recovery would have taken place if nothing at all had been done. Nor does it end here; in a short time his enthusiasm becomes so excited, that he prescribes it confidently to every body and for every thing. It was in this way that so great a man as Bishop Berkley, from having "improved" after taking some tar-water, advised others to take it, and in the end wrote a pamphlet advocating the use of tar-water as a valuable remedy in almost every form of disease. It somehow or other happened that when the Bishop was attacked with his final illness, there wasn't a spoonful of tar to be had "in all the region roundabout." To-day, an ocean of tar-water would not sell for two cents, as a remedy, to any sensible apothecary.

Within a month or two a gentleman had occasion to cross "the channel" between England and France, and, dreading sea-sickness very much, he wrapped himself up snugly in a corner on deck, took six or eight grains of chloral in half a glass of water, and went to sleep without having experienced any

SEA-SICKNESS.

Later on, in crossing the channel again, he took a similar dose under similar conditions. Many were made very sick; he was not.

It is not stated that he tried taking nothing, and escaped sea-sickness. It might have been a remedy for him, but not to others. Many will rush to the experiment of using it in crossing the Atlantic. But it might do for a few hours, and fail of all effect on the next day. Chloral is always uncertain and dangerous, and should be used only at the time of dissolving it, and even then a physician should be at hand to advise respecting it. Never take more than twenty grains at a dose; more powerful if in half a glass or more of water; take it before the vessel is in motion, but not before fixed, so as not to be disturbed or aroused. Lie down and remain on deck; it induces sleep or semi-consciousness. Do not repeat a twenty grain dose in less intervals than twelve hours on long voyages, and then five grain doses twice daily, until you become accustomed to the motion of the vessel.

